# Comparing health care workforce in circumpolar regions: patterns, trends and challenges

**DOI:** 10.1080/22423982.2018.1492825

**Published:** 2018-07-03

**Authors:** T. Kue Young, Natalia Fedkina, Susan Chatwood, Peter Bjerregaard

**Affiliations:** a School of Public Health, University of Alberta, Edmonton, Canada; b Institute for Circumpolar Health Research, Yellowknife, Northwest Territories, Canada; c Dalla Lana School of Public Health, University of Toronto, Toronto, Canada; d National Institute of Public Health, University of Southern Denmark, Copenhagen, Denmark; e Greenland Centre for Health Research, University of Greenland, Nuuk, Greenland

**Keywords:** Health workforce, physicians, dentists, nurses, Arctic, North, circumpolar

## Abstract

**Background:** The eight Arctic States exhibit substantial health disparities between their remote northernmost regions and the rest of the country. This study reports on the trends and patterns in the supply and distribution of physicians, dentists and nurses in these 8 countries and 25 regions and addresses issues of comparability, data gaps and policy implications

**Methods:** We accessed publicly available databases and performed three types of comparisons: (1) among the 8 Arctic States; (2) within each Arctic State, between the northern regions and the rest of the country; (3) among the 25 northern regions. The unit of comparison was density of health workers per 100,000 inhabitants, and the means of three 5-year periods from 2000 to 2014 were computed.

**Results:** The Nordic countries consistently exceed North America in the density of all three categories of health professionals, whereas Russia reports the highest density of physicians but among the lowest in terms of dentists and nurses.

The largest disparities between “north” and “south” are observed in the Northwest Territories and Nunavut of Canada for physicians, and in Greenland for all three categories. The disparity is much less pronounced in the northern regions of Nordic countries, while Arctic Russia tends to be oversupplied in all categories.

**Conclusions:** Despite efforts and standardisation of definitions by international organisations such as OECD, it is difficult to obtain an accurate and comparable estimate of the health workforce even in the basic categories of physicians, dentists and nurses . The use of head counts is particularly problematic in jurisdictions that rely on short-term visiting staff. Comparing statistics also needs to take into account the health care system, especially where primary health care is nurse-based.

**List of Abbreviations** ADA: American Dental Association; AHRF: Area Health Resource File; AMA: American Medical Association; AO: Autonomous Okrug; AVI: Aluehallintovirasto; CHA: Community Health Aide; CHR: Community Health Representative; CHW: Community Health Worker; CIHI: Canadian Institute for Health Information; DO: Doctor of Osteopathic Medicine; FTE: Full Time Equivalent; HPDB: Health Personnel Database; MD: Doctor of Medicine; NOMESCO: Nordic Medico-Statistical Committee; NOSOSCO: Nordic Social Statistical Committee; NOWBASE: Nordic Welfare Database; NWT: Northwest Territories; OECD: Organization for Economic Co-operation and Development; RN: Registered Nurse; SMDB: Scott’s Medical Database; WHO: World Health Organization

## Introduction

The health workforce is an important and critical “building block” of any health system. It needs to be responsive, fair and efficient given available resources, and accessible in sufficient quantity [1]. Developing, managing and sustaining the health workforce is a priority of most national health systems. A recent OECD review of health workforce policies found a continuing need to focus on addressing shortages, maldistribution and suboptimal skill mix [2]. Within countries, even those at a highly developed state of economic development, rural and remote communities tend to experience reduced access to health services. Redressing such inequality would require special health workforce policies that target these vulnerable populations [3].

Developing evidence-based health workforce policy and planning depends on the availability and completeness of quality data. WHO has identified the need to harmonise definitions and classifications of all health workers across sources, countries and time [4]. Deficiency in health workforce data is a problem faced not just by lower-income countries, but exists also in high-income countries, especially their remote regions.

This article presents statistics on the trends and patterns in the supply and distribution of physicians, dentists and nurses in the Arctic States and their northern regions, and addresses issues of comparability, data gaps and policy implications. The Arctic States refer to the eight member states of the intergovernmental Arctic Council, comprising Canada, Denmark (with its autonomous territories of Greenland and Faroe Islands), Finland, Iceland, Norway, Sweden, the Russian Federation and the United States. These countries are among the world’s most developed economies with a high standard of living and highly educated health workforce. However, within these countries, substantial health disparities continue to exist between their remote, northernmost regions and the rest of the country [5].

## Background

The health workforce is made up of many different types of workers. WHO’s *International Classification of Health Workers* recognises five broad categories: (1) health professionals, (2) health associate professionals, (3) personal care workers in health services, (4) health management and support personnel, and (5) other health service providers [6]. International comparisons of the size and distribution of the health workforce are beset by serious methodological challenges.

An obvious but perhaps least complicated issue in international comparison is language. While a dictionary can provide the equivalent of “doctor,” “nurse,” or “dentist” in any language, there are underlying contextual and conceptual differences in how these health workers are named and differentiated. Across nation-states, health professionals vary substantially in their training, licensure and registration requirements, and how statistics on employment are collected. For within-country, interregional comparisons, substantial variations in how the health workforce is deployed, employed and regulated offer additional obstacles.

**Figure 1.**
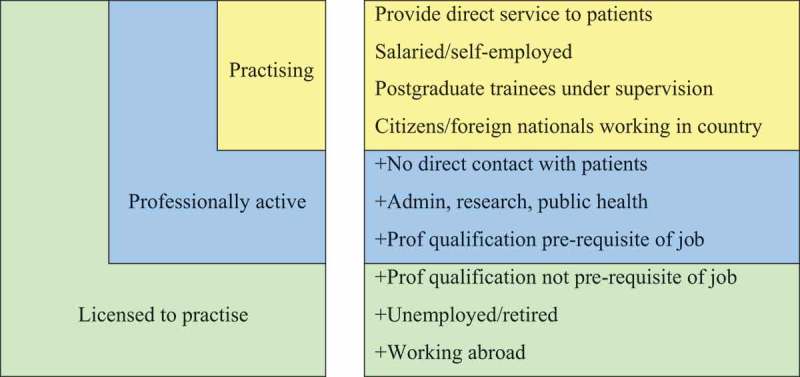
OECD classification of health professionals.

Since 2010, OECD’s health statistics database [7] has made the distinction among the categories of “practising” “professionally active,” and “licensed to practise” health workers. This is summarised in Figure 1. In earlier compendia of health data, the distinction was made only between “practising” and “licensed to practise” Some countries are not able to distinguish “practising” and “professionally active” – these are either left blank, or they provide identical numbers for the two categories.

There are usually two ways to count workers, by a head count or the number of full-time equivalent (FTE). It is difficult to apply the FTE concept to self-employed professionals such as physicians and dentists in some countries who do not have regular hours of work. Most data sources offer only head counts. Some jurisdictions provide data from employment records, some conduct surveys or censuses, while others include only public sector employees.

Trainees in medicine and dentistry, termed interns and residents in North America, who have graduated but are undergoing further clinical training under supervision, are generally counted as practicing physicians and dentists. Generalists and specialists can be further differentiated, although they are not considered in this paper. The United States is unique in including doctors of osteopathic medicine (DO) as physicians. DOs are now indistinguishable in training and scope of practice from doctors of medicine (MDs), whereas outside the U.S. osteopaths tend to treat only musculoskeletal disorders. Dentists (or stomatologists) are not included under physicians, although in some countries (such as Russia) they are considered medical specialists.

Under “professional nurses” OECD includes clinical nurses, district nurses, nurse-anaesthetists, nurse-educators, nurse-practitioners, public health nurses and specialist nurses. Excluded are midwives (unless they are also registered as nurses and working as nurses), nursing aides, associate professional nurses, practical and vocational nurses.

## Methods and data sources

We performed three types of comparisons: (1) comparing the eight Arctic States based on OECD data; (2) within each Arctic State, comparing its northern regions with the rest of the country using national data sources, with some exceptions explained below; and (3) comparing the northern regions. The unit of comparison was density of health workers in the population (per 100,000). The means of three 5-year periods from 2000 to 2014 were computed. Age-standardisation of rates is not appropriate as it is the crude rate that truly reflects the actual availability of health workers.

### Defining northern regions

We identified 25 northernmost regions within the 8 Arctic States (Table 1). The 25 regions include 1 American state, 3 Canadian territories, Greenland, Faroe Islands, Iceland, the northernmost “counties” in Norway (*fylke*), Sweden (*län*) and Finland (*lääni*), and various Russian administrative entities. These are “subnational” regions. Health data, including data on the workforce, are collected and reported by administrative regions and not by geophysical boundaries or community characteristics such as remoteness or population density. Our data can only be used for interregional but not intraregional comparisons.

**Table 1.** Circumpolar countries and regions.

Arctic state	Northern region
United States of America	Alaska
Canada	Yukon
	Northwest Territories
	Nunavut
Denmark	Greenland
	Faroe Islands
Iceland	Iceland
Norway	Nordland
	Troms
	Finnmark
Sweden	Västerbotten
	Norrbotten
Finland*	Pohjois-Suomi
	Lappi
Russian Federation**	Murmansk Oblast
	Kareliya Republic
	Arkhangelsk Oblast
	– Nenets AO
	Komi Republic
	Yamalo-Nenets AO
	Khanty-Mansi AO
	Krasnoyarsk Kray
	– Taymyr AO
	– Evenkia AO
	Sakha Republic
	Magadan Oblast
	Kamchatka Kray
	– Koryak AO
	Chukotka AO

Note: * Finland – Pohjois-Suomi AVI formerly Oulun lääni – see text ** Russia – Taymyr, Evenkia and Koryak AO lost their status as federal subjects in 2007 and data no longer reported – see text

In 2010, Finland abolished the *lääni* and replaced it with the regional state administrative agency (*aluehallintovirasto* or AVI). For the northern regions of Oulu (now called Pohjois-Suomi) and Lappi, there is little impact on boundaries. Some statistical data from Finland, however, are presented in lower-order administrative units such as the *maakunta* and hospital districts (*sairaanhoitopiiri*), but these can be aggregated into AVIs.

The Russian Federation is composed of different types of administrative units called federal “subjects” (*sub’ekty*), including republic, *kray, oblast*, autonomous *okrug*, and federal city, with varying degrees of autonomy. An autonomous okrug (AO), with the exception of Chukotka, is generally part of some higher level units such as an oblast or kray, and usually represents the traditional territory of some indigenous ethnic group. Statistical data are usually available for these AOs separately. As of 1 January 2007, Taymyr, Evenkia and Koryak AO ceased to exist as distinct federal subjects, and were fully absorbed into the Krasnoyarsk kray and Kamchatka kray, and statistics are no longer reported for these former AOs.

Iceland as a sovereign country is treated as both a nation-state when comparing with the other Arctic States, and also as a region when comparing with the other northern regions. There is no northern region within Iceland.

In the case of Greenland and Faroe Islands, which enjoy a high degree of self-government, we compare them to Denmark. [Denmark, Greenland and Faroe Islands together constitute the Kingdom of Denmark.] Statistics from Greenland and Faroe Islands are collected separately from and are not included in Danish national statistics. Since the population of Greenland is only about 1%, and that of the Faroe Islands about 0.8%, of that of Denmark, creating a “Kingdom of Denmark” dataset by combining data from Denmark, Greenland and the Faroe Islands would be virtually identical to that of Denmark alone. Comparing Greenland and Faroe Islands with Denmark is also justified in that the health care system in the two self-governing regions is modelled after that of Denmark, and most health care professionals receive their training in Denmark.

In the other countries except Iceland, the northern regions are compared to the country as a whole. Statistics on these northern regions are part of national statistics.

### International databases

Seven of the eight Arctic States are among the 35 members of the Organization for Economic Co-operation and Development (OECD). A variety of health and social statistics from these seven Arctic States are included in the *OECD Health Statistics* database [7]. Although the Russian Federation is not a member of OECD, some Russian statistics (such as data on physicians and nurses) are also reported by OECD for comparison.

The Nordic countries are members of the Nordic Medico-Statistical Committee (NOMESCO) and the Nordic Social Statistical Committee (NOSOSCO), and they contribute statistics to the Nordic Welfare Data Base (NOWBASE) [8]. Data for Greenland and Faroe Islands are presented separately. Health workforce data are among the health care statistics available. NOMESCO also produces the annual report *Health Statistics for the Nordic Countries* [9].

The World Health Organization (WHO) also reports on health workforce statistics, which can be accessed through its Global Health Observatory [10]. However, there are many gaps in many national data series, even for the highly developed Arctic States, and there is scant documentation on the data sources for each country. We have not utilised data from WHO in this study.

Different data sources do not agree completely on the number of health workers for the same countries in the same year. When expressed as rates and averaged over 5-year periods, these discrepancies are minor and do not affect the relative ranking of countries or regions.

### USA

There is no single source of information on health human resources. For physicians, the source is the American Medical Association’s *Physicians Masterfil*e as reported in the *Area Health Resource File* (AHRF) produced by the Bureau of Health Professions, Health Resources and Services Administration (HRSA), Department of Health and Human Services [11]. A record in the AMA Masterfile is created as a physician is licensed in a U.S. jurisdiction or begins medical school or residency training. It is then continuously updated with new information on career path, professional certifications and medical practice characteristics.

The American Dental Association (ADA) conducts an annual census of all known dentists in the United States. Data for 2000–2008 were extracted from the ADA publication *Distribution of Dentists* for 1998–2006 [12], 2007 [13] and 2008 [14]. After 2009 data on dentists are available also from the AHRF [11]. Professionally active dentists include those engaged in dentally related activities such as education, research and clinical practice, either as a primary or secondary occupation.

Data on nurses are obtained from the Occupational Employment Statistics database of the Bureau of Labor Statistics [15]. However, only salaried employees of health care institutions are included. For 2000–2011, one category of “registered nurses” (code 29–111) was reported. From 2012 on, the category of “registered nurses” (code 29–141) is reported separately from, and does not include nurse-anaesthetists (29–1151), nurse-midwives (29–1161), and nurse-practitioners (29–1170).

The HRSA has also conducted surveys of the population of registered nurses every 4 years between 1977 and 2008, which contains more details on the workforce, and can identify those who are specifically employed in nursing among the total employed population as well as distinguish part-time from full time [16].

### Canada

Data on physicians are from the data tables in the report *Supply, Distribution and Migration of Canadian Physicians*, annually updated by the Canadian Institute for Health Information (CIHI). Data for 2000–2010 were from the 2010 report [17] and data for 2010–2014 from the 2015 report [18]. CIHI maintains the *Scott’s Medical Database* (SMDB), derived from a commercial firm that produces the *Canadian Medical Directory* and mailing lists targeting physicians in the country. SMDB produces headcounts of “active” physicians, defined as individuals with a medical degree who have a valid mailing address. Interns and residents are excluded, as are military, nonregistered and semiretired physicians, but physicians not engaged in clinical practice (e.g. education, administration and research) are included [19].

OECD is able to derive an estimate of *practising* physicians for Canada from CIHI’s estimate of *professionally active* physicians by multiplying the latter with the proportion of physicians who report that they provide patient care in their response to the National Physician Survey. This proportion is about 95%. It further adds the number of interns and residents from the Canadian Post-MD Education Registry [20].

Data on dentists for 2000–2011 are from CIHI’s Health Personnel Database (HPDB) and included in the periodic publication *Canada’s Health Care Providers* [21]. Data from the Northwest Territories (NWT) and Nunavut were combined before 2004. Data for 2012 and 2013 were specially requested from CIHI in October 2016.

Data on nurses are available from the CIHI report *Canada’s Health Care Providers* [21] for 2000–2005, and *Regulated Nurses* [22] for 2006–2014. The source for both reports is the HPDB. CIHI distinguishes “supply” from “workforce.” The former refers to nurses who are eligible to practise in the given year, whether employed or not employed at the time of registration; the latter is a subset of supply, referring to only those who are employed at the time of registration [23]. While data on registered nurses (RNs), licensed practical nurses and registered psychiatric nurses are provided by CIHI, only RNs are considered in this study.

NWT and Nunavut data are combined for nurses. Only registered nurses employed in nursing (including administration and research) are included. While nurse-practitioners are employed in the territories, these individuals are also registered nurses.

Note that the CIHI data for dentists and nurses refer to professionals registered to practise in the territories, who may not be resident in the territories and are only engaged in part-time practice consisting of multiple short visits.

### Denmark, Greenland and Faroe Islands

We compared Greenland and Faroe Islands with Denmark, using data from NOMESCO’s *Health Statistics in the Nordic Countries* [9] and NOWBASE [8], its social and health indicators database. NOMESCO obtains workforce data for Greenland from the Office of the Chief Medical Officer (*Landslægeembedet*), and for Faroe Islands from its Ministry of Health and the Interior (*Heilsu-og innlendismálaráđiđ*). For Denmark, since 2015 NOMESCO has received data from the *Sundhedsdatastyrelsen*, a central repository of health status and health care data. Prior to that, health workforce data were accessed from the *Statens Serum Institut* or directly from the Danish Health Authority (*Sundhedsstyrelsen*).

Note that physicians and dentists in Greenland and the Faroe Islands are registered and regulated by the Danish Patient Safety Authority (*Styrelsen for Patientsikkerhed)*. Nurses in the Faroe Islands are also regulated by Denmark but in Greenland they are under the responsibility of the Chief Medical Officer. Because of the lack of registered nurses, Greenland has for many years deployed “health aides” (*sundhedsmedhjælper*), who have a 3 years’ training in Greenland and who in many ways perform the work of nurses. They are the backbone of the Greenland health care system but are gradually being replaced by locally trained professional nurses.

### Iceland

Iceland’s data are taken from OECD. Physician and dentist data originated from the registers of physicians and dentists maintained by the Directorate of Health (*Embætti landlæknis*). Data on professional nurses are based on membership in the Icelandic Nurses Association (*Félag íslenskra hjúkrunarfræðinga*).

### Norway, Sweden and Finland

Data are from Statistics Norway’s Statbank tables [24]. Data on the health workforce are derived from the register of health care personnel administered by the Directorate of Health (*Helsedirektoratets helsepersonellregister*). Health professionals refer to “persons with health care education employed in region.” For 2000–2007, data refer to individuals aged 16–66, for 2008 and thereafter, the age group is 15–74. Nurses include public health nurses. Physicians include both generalists and specialists.

Swedish national and regional data are from the National Board of Health and Welfare (*Socialstyrelsen*) database [25]. Nurses include public health nurses.

Finnish data are from SOTKAnet [26], operated by the National Institute of Health and Welfare (*Terveyden ja hyvinvoinnin laitos*, THL). For 2000–2007, data on only those employed by municipal health services were reported. Only dentists employed in primary care were included. A new series which includes both private and public sector employees was introduced in 2010. Nurses include public health nurses in both series. Midwives are included with nurses after 2010.

### Russia

Data are as reported in the biennial report *Health Care in Russia* (*Zdravookhranenie v Rossii*) [27] published by the Federal State Statistics Service (*Federal’naia sluzhba gosudarstvennoi’ Statistiki*, or Rosstat).

Included under “dentists” are stomatologists (*stomatologi*) but not middle-level dentists (*zubnye vrachi*). The number of stomatologists, however, is included with physicians (*vrach*) and they have therefore been deducted from the total number of physicians. Nurses (*medicinskie sestry*) and midwives (*akusherki*) are middle-level health staff who, together with various health care technicians and assistants, are referred to as supporting medical personnel. No data are reported from Taymyr, Evenkia, Koryak AO after 2007.

*Health Care in Russia* is published only in alternate years and covers only 1 year of data. Thus in Table 7, the 2000–2004 period refers to the mean of 2000 and 2004; the 2005–2009 period the mean of 2006 and 2008; and the 2010–2014 period the mean of 2010, 2012 and 2014.

## Results

We present descriptive statistics in tables and graphs. Due to comparability issues, discussed later, we have not performed inferential statistics when making the various types of comparisons.

### National comparisons

For comparisons of the eight Arctic States, we used the data from OECD, which attempts to achieve consistency in definitions. We reported on “practising” professionals for the period 2010–2014 (Table 2). Where they are not available, data for “professionally active” personnel are used. Although OECD presents Russian physician data which originated from Rosstat, the series includes stomatologists. We have subtracted the number of stomatologists from the total number of physicians.

**Table 2.** Ranking of health professional densities in the Arctic States, 2010–2014.

Physicians	Density	Dentists	Density	Nurses	Density
Russia	456	Norway	87	Norway	1652
Norway	425	Iceland	87	Sweden	1114
Sweden	405	Sweden	81	Denmark	981
Denmark	364	Denmark	78	Finland	964
Iceland	358	Finland	75	Iceland	894
Finland	308	Canada	61	USA	881
Canada	248	USA	60	Russia	738
USA	251	Russia	43	Canada	698

Sources: OECD for all countries except Russia; Russia data from Roostat. See text for details Notes: Data refer to “practicing” health professionals except for dentists in the United States and Canada and nurses in the United States, which refer to “professionally active.”

Russia reports the highest density of physicians, while Canada and the United States occupy the low end, with the Nordic countries in the intermediate ranks. Canada and the United States report low densities also for dentists and nurses. In contrast to physicians, Russia reports low densities for dentists and nurses. The Nordic countries consistently exceed North America in the density of all three categories of health professionals.

### North-South comparisons

Table 3 to Table 6 compare the density of physicians, dentists and professional nurses in circumpolar countries and regions. Where available, the data for “practising” professionals are shown, except where they are not distinguishable from the “professionally active.” Note that data for nation-states are slightly different from those reported in Table 3 based on OECD.

**Table 3.** Density of health professionals (per 100,000) in North America.

	2000–2004	Ratio	2005–2009	Ratio	2010–2014*	Ratio
**Physicians**						
USA	280	1.00	289	1.00	299	1.00
Alaska	230	0.82	248	0.86	255	0.85
Canada	189	1.00	195	1.00	216	1.00
Yukon	171	0.91	218	1.12	194	0.90
Northwest Territories	107	0.57	107	0.55	89	0.41
Nunavut	29	0.15	36	0.19	36	0.17
**Dentists**						
USA	59	1.00	60	1.00	60	1.00
Alaska	73	1.22	75	1.26	78	1.30
Canada	57	1.00	59	1.00	60	1.00
Yukon	81	1.42	106	1.80	118	1.88
Northwest Territories	] 64	] 1.12	115	1.95	127	1.07
Nunavut			142	2.41	209	1.61
**Nurses**						
USA	779	1.00	822	1.00	880	1.00
Alaska	766	0.98	758	0.92	788	0.90
Canada	755	1.00	785	1.00	787	1.00
Yukon	909	1.20	1007	1.28	1061	1.35
Northwest Territories	] 1101	] 1.46	1413	1.80	1410	1.79
Nunavut						

Notes: * 2014 or most recently available data. Sources: Canadian data from CIHI; USA data from AHRF. See text for details. USA data for the period 2000–2009 are from the 2010/12 vintage AHRF datafiles accessed in 2013 and data for 2010–2014 are from the 2015/16 vintage datafiles accessed in 2016.

**Table 4.** Density of health professionals (per 100,000) in Denmark, Greenland, Faroe Islands and Iceland.

	2000–2004	Ratio	2005–2009	Ratio	2010–2014*	Ratio
**Physicians**						
Denmark	298	1.00	336	1.00	358	1.00
Greenland	148	0.50	178	0.53	171	0.48
Faroe Islands	192	0.64	188	0.56	245	0.67
Iceland	354	1.00	362	1.00	358	1.00
**Dentists**						
Denmark	85	1.00	84	1.00	78	1.00
Greenland	53	0.63	50	0.60	48	0.62
Faroe Islands	82	0.96	83	0.99	88	1.12
Iceland	100	1.00	94	1.00	87	1.00
**Nurses**						
Denmark	963	1.00	974	1.00	1014	1.00
Greenland	397	0.41	473	0.49	448	0.44
Faroe Islands	756	0.79	603	0.62	906	0.89
Iceland	829	1.00	855	1.00	894	1.00

Notes: * 2014 or most recently available data. Sources: Data for Denmark, Greenland and Faroe Islands are from NOMESCO. Data for Iceland are from OECD. See text for details.

**Table 5.** Density of health professionals (per 100,000) in Norway, Sweden and Finland.

	2000–2004	Ratio	2005–2009	Ratio	2010–2014*	Ratio
**Physicians**						
Norway	359	1.00	427	1.00	487	1.00
Nordland	309	0.86	375	0.88	453	0.93
Troms	539	1.50	662	1.55	746	1.53
Finnmark	336	0.94	393	0.92	490	1.01
Sweden	329	1.00	368	1.00	405	1.00
Västerbotten	403	1.23	447	1.22	508	1.25
Norrbotten	243	0.74	267	0.72	292	0.72
Finland	217	1.00	226	1.00	305	1.00
Oulu	259	1.19	263	1.16	336	1.10
Lappi	158	0.73	181	0.80	213	0.70
**Dentists**						
Norway	85	1.00	92	1.00	98	1.00
Nordland	70	0.82	83	0.90	95	0.97
Troms	89	1.05	100	1.09	124	1.27
Finnmark	71	0.84	76	0.83	84	0.86
Sweden	82	1.00	81	1.00	81	1.00
Västerbotten	91	1.12	85	1.05	89	1.10
Norrbotten	76	0.93	76	0.93	83	1.03
Finland	43	1.00	42	1.00	73	1.00
Pohjois-Suomi	53	1.23	55	1.31	82	1.12
Lappi	48	1.12	47	1.12	70	0.96
**Nurses**						
Norway	1596	1.00	1830	1.00	1971	1.00
Nordland	1502	0.94	1835	1.01	2106	1.07
Troms	1875	1.17	2449	1.34	2422	1.23
Finnmark	1495	0.94	1685	0.92	1965	1.00
Sweden	1024	1.00	1093	1.00	1113	1.00
Västerbotten	1270	1.24	1354	1.24	1396	1.25
Norrbotten	1054	1.03	1111	1.02	1147	1.03
Finland	671	1.00	757	1.00	981	1.00
Oulu	749	1.12	858	1.13	1085	1.11
Lappi	669	1.00	781	1.03	993	1.01

Notes: * 2014 or most recently available data. Sources: Data for Norway are from Statistics Norway, Sweden from Socialstyrelsen and Finland from SOTKANet. See text for details.

**Table 6.** Density of health professionals (per 100,000) in Russia.

	2000–2004	Ratio	2005–2009	Ratio	2010–2014*	Ratio
**Physicians**						
Russian Federation	431	1.00	451	1.00	456	1.00
Murmansk Oblast	438	1.02	433	0.96	477	1.05
Kareliya Republic	477	1.11	477	1.06	448	0.98
Arkhangelsk Oblast	448	1.04	469	1.04	467	1.02
– Nenets AO	321	0.74	433	0.96	428	0.94
Komi Republic	383	0.89	417	0.92	405	0.89
Yamalo-Nenets AO	434	1.01	441	0.98	445	0.98
Khanty-Mansi AO	409	0.95	470	1.04	517	1.14
Krasnoyarsk Kray	435	1.01	450	1.02	462	1.01
– Taymyr AO	471	1.09	478	1.06	Not available	
– Evenki AO	527	1.22	549	1.22	Not available	
Sakha Republic	449	1.04	508	1.13	533	1.17
Magadan Oblast	524	1.22	508	1.13	507	1.11
Kamchatka Kray	464	1.08	473	1.05	467	1.03
– Koryak AO	543	1.26	393	0.87	Not available	
Chukotka AO	584	1.35	723	1.60	668	1.47
**Dentists**						
Russian Federation	40	1.00	44	1.00	43	1.00
Murmansk Oblast	43	1.08	42	0.95	47	1.10
Kareliya Republic	17	0.42	14	0.33	22	0.51
Arkhangelsk Oblast	62	1.53	64	1.46	62	1.45
– Nenets AO	40	0.99	43	0.98	47	1.09
Komi Republic	42	1.04	41	0.95	39	0.90
Yamalo-Nenets AO	38	0.95	42	0.96	43	0.99
Khanty-Mansi AO	41	1.03	51	1.16	51	1.17
Krasnoyarsk Kray	42	1.05	51	1.17	49	1.14
– Taymyr AO	44	1.09	39	0.89	Not available	
– Evenki AO	34	0.84	35	0.80	Not available	
Sakha Republic	28	0.70	34	0.79	36	0.83
Magadan Oblast	37	0.92	30	0.70	36	0.84
Kamchatka Kray	39	0.96	42	0.96	40	0.94
Koryak AO	46	1.14	44	1.00	Not available	
Chukotka AO	60	1.49	74	1.69	64	1.48
**Nurses**						
Russian Federation	709	1.00	738	1.00	738	1.00
Murmansk Oblast	923	1.30	941	1.27	1050	1.42
Kareliya Republic	829	1.17	836	1.13	859	1.16
Arkhangelsk Oblast	918	1.29	978	1.32	992	1.34
– Nenets AO	590	0.83	695	0.94	822	1.11
Komi Republic	882	1.24	916	1.24	987	1.34
Yamalo-Nenets AO	883	1.24	901	1.22	915	1.24
Khanty-Mansi AO	890	1.26	1002	1.36	1054	1.43
Krasnoyarsk Kray	718	1.01	779	1.06	840	1.14
– Taymyr AO	740	1.04	848	1.15	Not available	
– Evenki AO	423	0.60	613	0.83	Not available	
Sakha Republic	862	1.22	892	1.21	930	1.26
Magadan Oblast	883	1.24	999	1.35	1040	1.41
Kamchatka Kray	833	1.17	857	1.16	869	1.18
– Koryak AO	882	1.24	878	1.19	Not available	
Chukotka AO	846	1.19	1063	1.44	1045	1.42

Notes: 2000–2004 – mean of 2000 and 2004 2005–2009 – mean of 2006 and 2008 2010–2014 – mean of 2010, 2012 and 2014 Source: Rosstat. See text for details

The overall pattern of disparities between “north” and “south” is far from uniform. The largest disparities between “north” and “south” are observed in the Northwest Territories and Nunavut of Canada for physicians. In the Kingdom of Denmark there are generally fewer physicians, dentists and nurses in Greenland and the Faroe Islands than in Denmark, with Greenland as the most disadvantaged part of the kingdom. The disparity is much less pronounced in the Nordic countries, with the “worst” ratio (0.7) observed among physicians in Norrbotten in Sweden and Lappi in Finland. In Russia, its northern regions tend to be oversupplied relative to the whole country in all three categories of providers.

In terms of trend over the three 5-year periods, the northern regions in the Nordic countries show a steady increase over time, whereas little increase (and even decline) can be observed in Alaska and northern Canada. The apparent increase in dentists in northern Canada is explained below. The pattern in the Russian regions is more mixed, with both increases and decreases.

### Inter-regional comparisons

**Figure 2.**
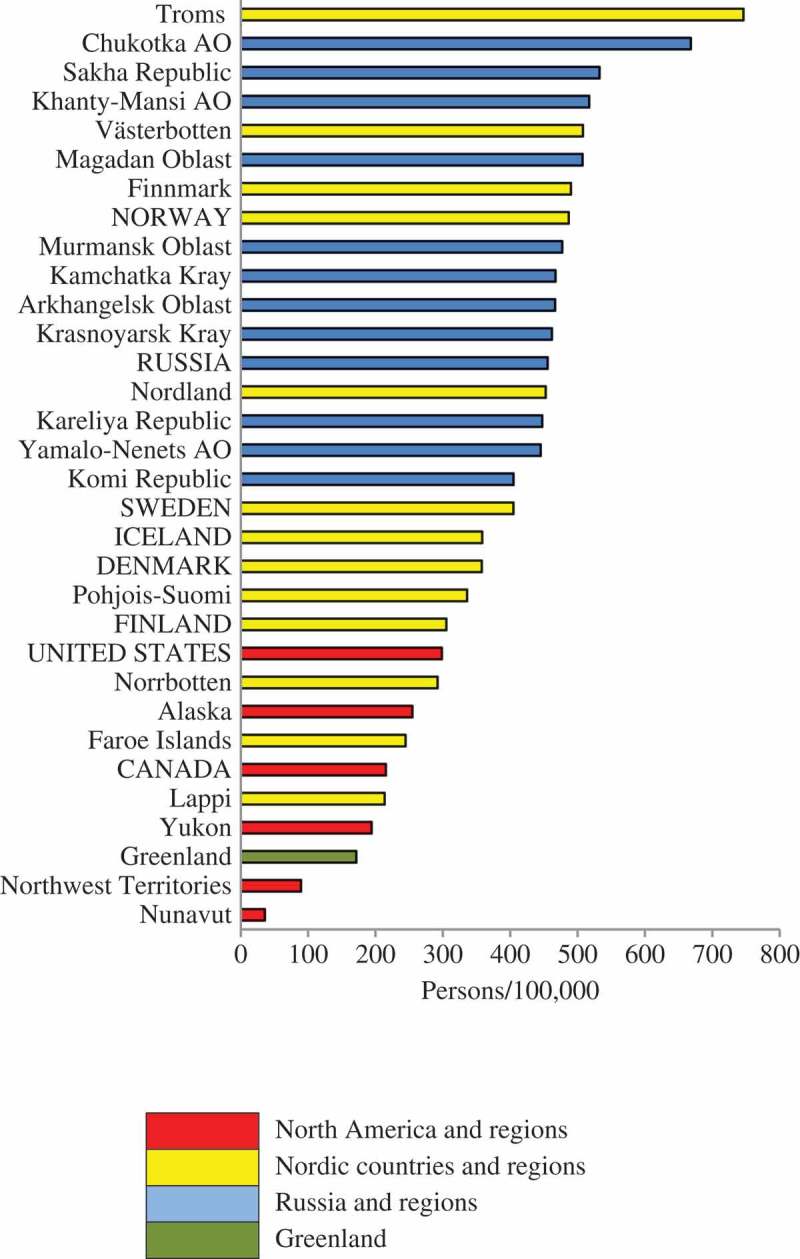
Density of practicing physicians (per 100,000) in eight Arctic States and their northern regions.

Figure 2 to Figure 4 rank the Arctic States and the 25 northern regions in terms of the density of physicians, dentists and nurses during the 2010–2014 period. In the colour-coding scheme, Denmark, Iceland and Faroe Islands are coded as Nordic countries/regions because of their shared history and demography. Greenland is given a separate colour code.

**Figure 3.**
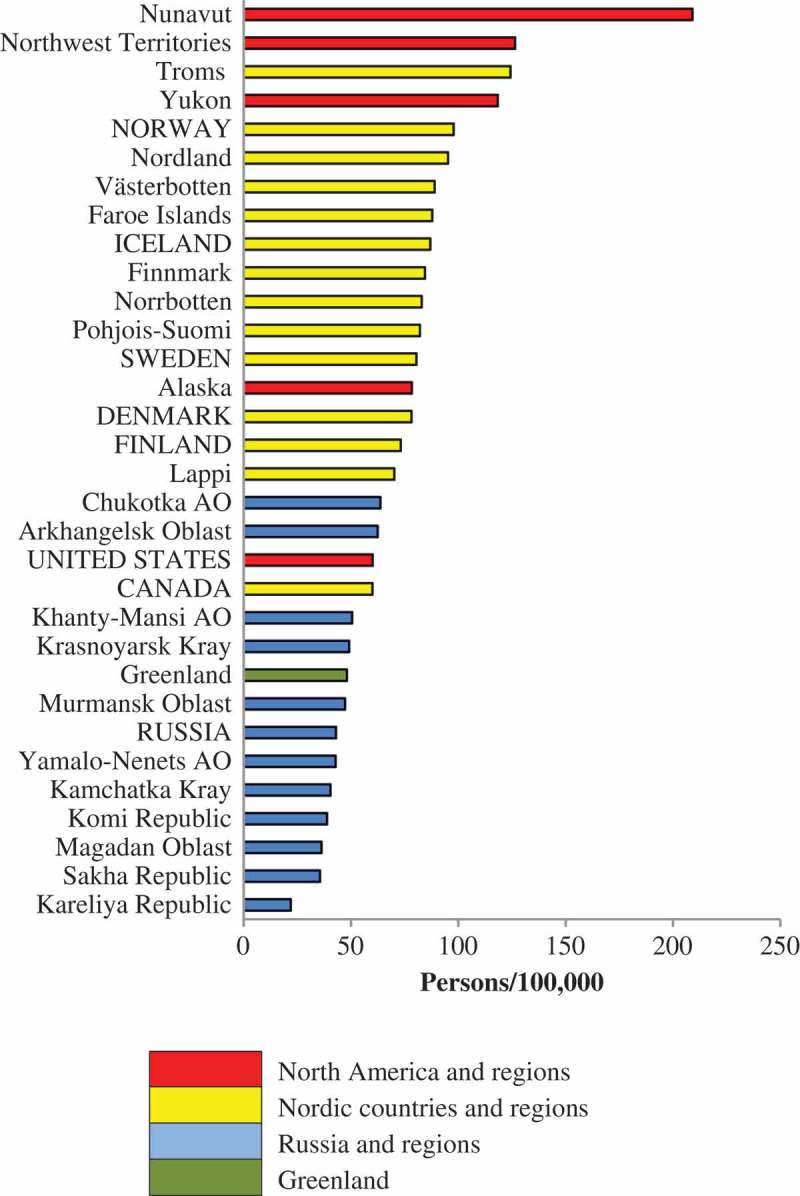
Density of practicing dentists (per 100,000) in eight Arctic States and their northern regions.

**Figure 4.**
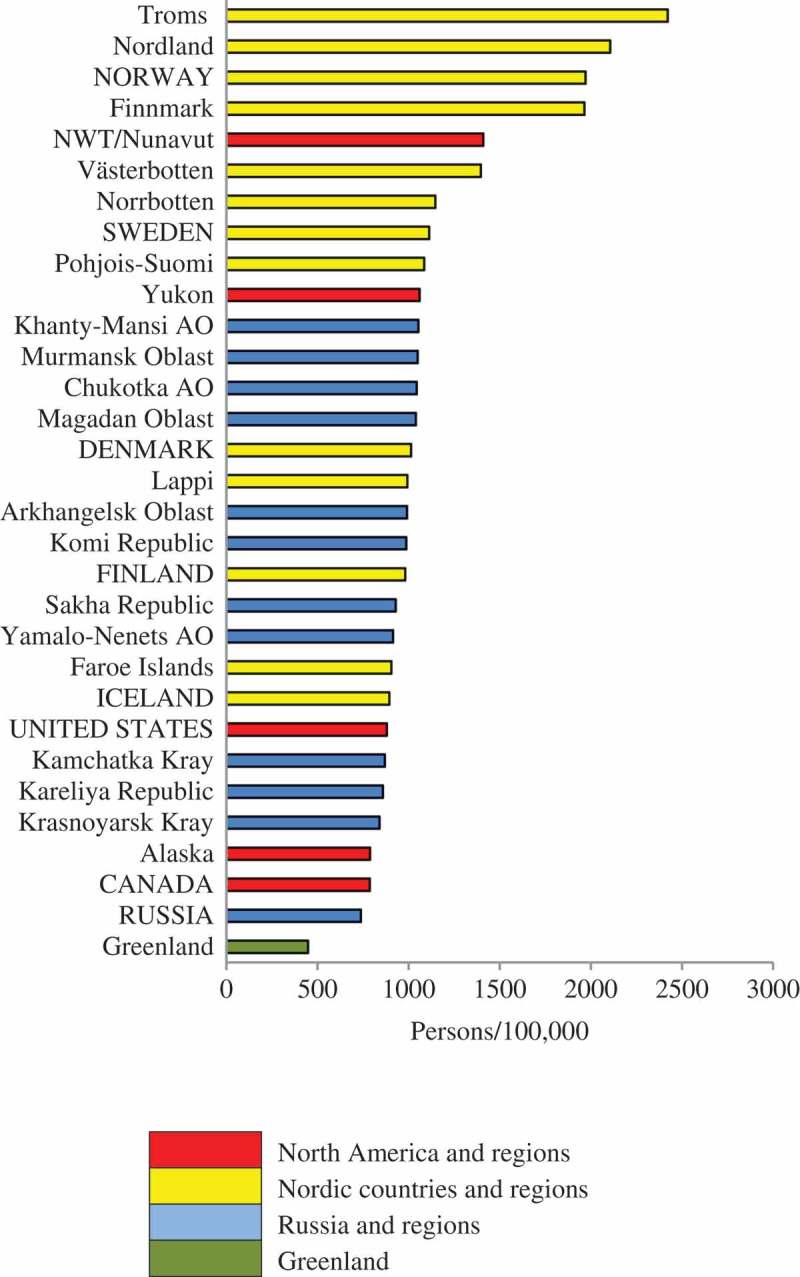
Density of practicing professional nurses (per 100,000) in eight Arctic States and their northern regions.

Northwest Territories, Nunavut and Greenland are similar in terms of physician density. There is clear separation between Russian regions and the northern regions of the Nordic countries, especially for physicians. The situation for dentists and nurses is more complex, as will be explained in the following text.

## Discussion

### Major patterns and trends

While Canada’s northern territories and Greenland have far lower density of physicians than Canada and Denmark, this is not the case in the Nordic countries or Russia, where some northern regions actually exceed the national norms. For nurses, the density in the Canadian North is substantially higher than that for Canada nationally because of the nature of the system that is predominantly nurse-based, with nurses practising in an expanded role. The density of nurses in Greenland is very low due to the fact that, for many years, Inuit health aides with 3 years’ formal education performed the work of registered nurses, but they are not counted as nurses. For the other regions, there is no consistency in terms of a northern deficit or excess. The density for nurses also tend to be high in the northern regions of Norway and Sweden.

The dentist situation is unique in Canada, where its 3 northern regions rank at or near the top among the 25 regions, a reflection of the use of short-term dentists which inflates the head count. While there are resident dentists in private practice in the larger cities in the Canadian North, the territorial governments contract with private practitioners across Canada to provide short-term visits to the remote communities.

The Russian health care system has a tradition of overcapacity from Soviet times under the Semashko model [28]. Despite the economic and political upheavals of the 1990s, physician density remains the highest in Europe. While there had been extensive out-migration of population (including health professionals) from the Arctic regions after the dissolution of the Soviet Union, some of these regions continue to have a much higher number of physicians per capita than the national average.

### Methodological challenges

We have provided a very detailed description of the various data sources to highlight their heterogeneity and the need for caution in comparing across nation-states and their northern regions. Over a 15-year period, there are breaks in data series with changes in categories and coverage.

Beyond the generic problem of headcounts not representing the true workforce available, numbers (and densities) for the Canadian North also fail to take into account the short-term stints of service by a large number of individuals. For example, in 2014, SMDB identified 12 physicians with a postal code in Nunavut (11 family physicians and 1 specialist). In reality, according to the territorial government, there were 157 individual family physicians employed/contracted by the Department of Health and Social Services, who together provided 7470 days of service in the territory, or 48 service-days per physician [19]. For dentists and nurses, on the other hand, CIHI headcounts are based on number of professionals registered/licensed and thus overestimate the supply.

It is clear that, even in highly developed economies such as the Arctic States, the current hodgepodge of approaches to obtain an estimate of the size of the health workforce in terms of even the basic categories of physicians, dentists and nurses is far from satisfactory. There is a need for more refined studies that investigate and develop comparable metrics, for example, “physician-days [or hours] in community” to assess accessibility to health professionals that are employed to work in the region and who travel to different communities within the region.

### Policy implications

Issues of health workforce recruitment and retention in remote communities have long occupied the attention of policy makers. Different countries and regions have designed incentive plans with varying degrees of success. It should be recognised that northern jurisdictions are not always trying to catch up with the rest of the country, but have in fact developed innovations that hold promise for jurisdictions beyond the North.

“Repatriating” health professional training to the North is one approach, on the assumption that students from the North are more inclined to study closer to home, and they are more likely to stay in the North after graduation. The Nordic countries, Russia and Alaska have a longer tradition of universities located in the North, for example, the University of Tromsø in Norway (69°N), Northern State Medical University in Arkhangelsk, Russia (64°N) and the University of Alaska Anchorage (61°N). The University of Greenland in Nuuk graduated its first nurses in 2010. In Canada, northern community colleges have agreements with southern universities for on-site nurses’ training resulting in the granting of degrees from those universities. For most regions, the supply of physicians and dentists will for many years depends on recruitment from outside the North.

There is a dearth of rigorous evaluation of recruitment and retention strategies in northern jurisdictions. A study in four remote Alaska Native health regions computed “survival” curves for three categories of practitioners – community health aides (CHA), nurses and physicians. It shows that CHAs, mostly recruited from among the communities where they served, had the best retention record, compared to physicians and nurses, whose median length of stay was less than 2 years [29]. It should be noted that physicians and nurses, even those from the communities who have returned to serve, have substantially more national mobility than CHAs.

Technology innovation can relieve or potentially even eliminate health human resources shortages. Of particular promise is the use of remote-presence devices in providing acute care, chronic disease management and continuing education [30]. In Labrador, Canada, the use of a roving robot in a remote community connected to a physician in the regional hospital has been shown to be well accepted by providers and patients, and have reduced the cost of aeromedical evacuations [31]. More broadly, telehealth or eHealth systems have spawned a huge literature worldwide, and there is no shortage of pilot or demonstration projects in the North [32].

The content and context of the work of a health care provider in the North is as important as the number of providers. Nursing practice in Greenland and other remote regions demands a wide range of skills which span the spectrum of primary care, public health and psychosocial health services [33]. While “task shifting” – the redistribution of tasks among different categories of providers – may be a relatively new term [34], the concept and practice have indeed been in place in the North for decades. The Alaska Native health system has pioneered the training and deployment of community health aides [35], some of whom are specialised to work in areas such as dental care [36]. In Canada, the use of community health representatives (CHR) or workers (CHW) as providers of first-contact care has declined in recent years, as most communities are now staffed by nurses, while CHRs/CHWs are now used mainly for prevention and health promotion work. In Greenland, health aides have for years performed the work of registered nurses. The health reform in Greenland in 2011 witnessed the start of a consolidation of small hospitals staffed by physicians in small towns to larger regional centres, who are replaced by nurses providing primary care.

Quality is just as important as quantity in the health workforce, and this is particularly true for cultural and language competence. In regions where Indigenous people predominate or constitute a substantial minority, health professional staff tends to be short-term visitors from outside the North whose knowledge of local conditions is often limited and clinical communication is filtered through interpreters. The high turnover of staff compromises continuity of care and the immediate needs of acute care lead to the relative neglect of prevention and health promotion activities.

It is important to recognise that the “North” is far from homogeneous. There are very large urban centres with populations over 100,000, especially in Russia and the Nordic countries. Even in northern Canada and Greenland, where their largest cities have fewer than 25,000 residents, urban health care is not substantially different than national norms, and the shortage of health workforce much less acute than in the remote communities. Road access is widely available in the Nordic countries, where distances from regional referral centres are much shorter, there is much less dependence on expensive air travel for patients and staff.

This paper compares one aspect of health care – the health workforce – in the northern regions of the eight Arctic States. These are among the world’s economically most advanced countries, where substantial financial resources are available to improve health care. The major rationale for making circumpolar comparisons is that collaboration across northern regions and the sharing of best practices offer an effective approach to health system improvement [37,38]. For this to be realised, significant evidence gaps such as those relating to the health workforce need to be closed. There is a need for a circumpolar working group on health workforce development which includes a data harmonisation component, perhaps in collaboration with national and international statistical agencies.
